# P-2020. Community Acquired Pneumonia Dashboard: An Antimicrobial Stewardship Solution to Treatment Guidance Adherence Monitoring

**DOI:** 10.1093/ofid/ofaf695.2184

**Published:** 2026-01-11

**Authors:** Daniel Rogers, Sahand Golpayegany, Julianne Gent, Sujit Suchindran, Lucy S Witt, Darshan Patel, K Ashley Jones

**Affiliations:** Emory Decatur Hospital, Lawrenceville, GA; Emory Healthcare, Marietta, Georgia; Emory Healthcare, Marietta, Georgia; Emory University School of Medicine, Atlanta, GA; Emory University, Atlanta, Georgia; Emory Johns Creek Hospital, Acworth, GA; Emory Healthcare, Marietta, Georgia

## Abstract

**Background:**

Centers for Disease Control and Prevention Core Elements of Hospital Antibiotic Stewardship Programs and Joint Commission standards charge antimicrobial stewardship programs (ASPs) with evaluating adherence to institution-specific treatment guidance. Adherence is commonly assessed via medication use evaluations, which are time and resource intensive and only provide data for a defined time period. Here we describe our approach to developing a fully automated dashboard to perpetually monitor adherence to our institution’s community acquired pneumonia (CAP) guidance across a large healthcare system.

**Figure:**
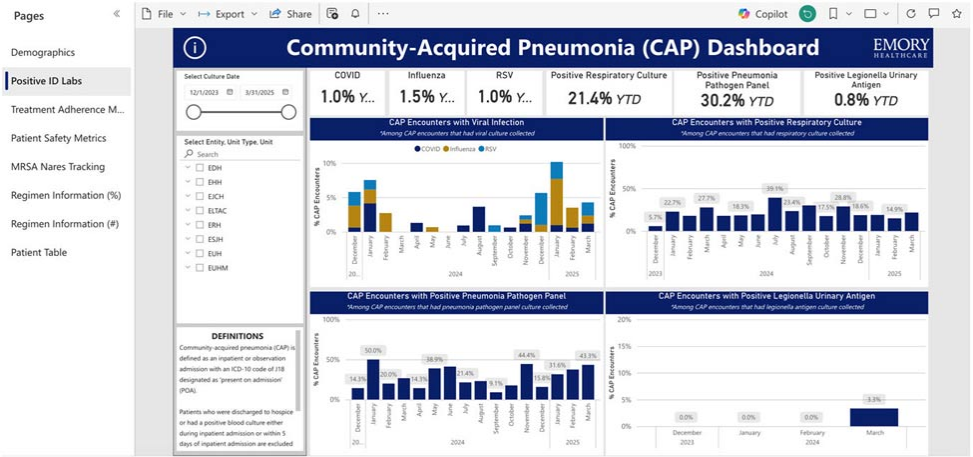
The “Positive ID Labs” Section This section shows any positive diagnostic data for the included patients.

**Figure:**
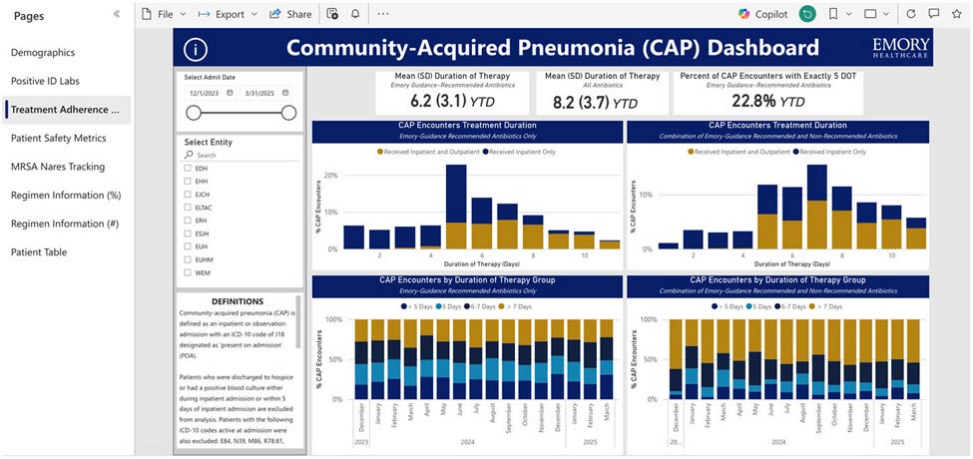
The “Treatment Adherence Metrics” Section This section shows the data relating to inpatient and outpatient durations of therapy, treatment courses including only institutional guidance recommended antimicrobials, and treatment courses including antimicrobials not recommended by institutional guidance.

**Methods:**

We used electronic medical record SQL queries to identify both inpatient and outpatient antimicrobials prescribed to adults (≥18 years) diagnosed with CAP and admitted to our healthcare system. Inpatient antimicrobials were based on bar-code administration and outpatient antimicrobials were captured from the discharge summary. The data were cleaned and analyzed in R Studio and visualized into a dashboard via PowerBI. The dashboard organizes the data into sections and graphics deemed appropriate by a multidisciplinary group of infectious diseases (ID) physicians, ID pharmacists, and a data analyst. Data updates are automated and visualizations can be easily revised as desired by our ASP.

**Figure:**
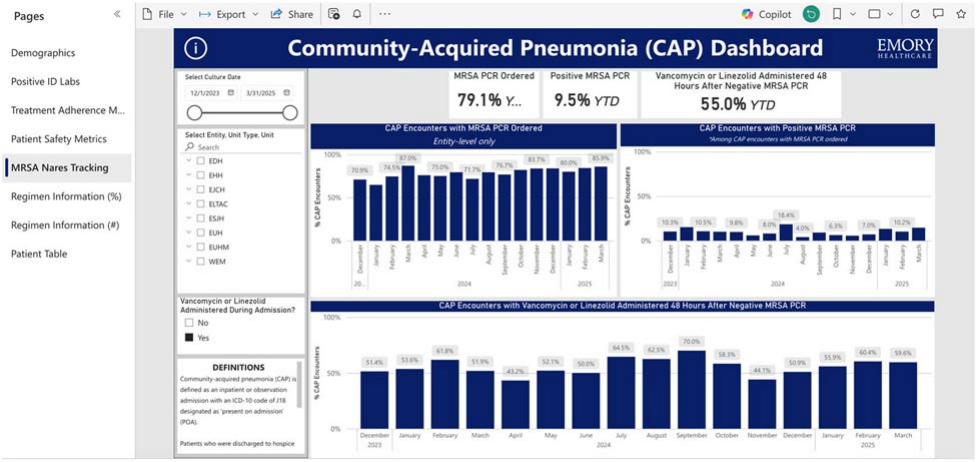
The “MRSA Nares Tracking” Section This section is designed to assess utilization and adherence to our MRSA nares screening. Note the option to filter the data to display all included patients or only those administered vancomycin or linezolid during admission.

**Figure:**
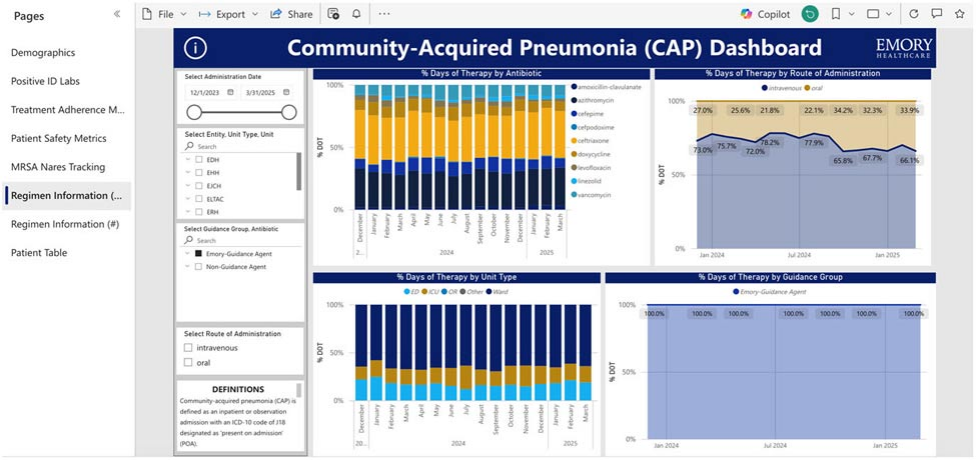
The “Regimen Information (%)” Section This section displays percentages of the overall days of therapy attributed to certain antimicrobials and routes of administration. Note the options to filter by entity, guidance agents vs. non-guidance agents, and route of administration.

**Results:**

We successfully created a dashboard to assess adherence to institutional treatment guidance and key ASP concerns such as duration of therapy, antimicrobial selection, and route of administration. Sections include demographics, positive ID labs, treatment adherence metrics, patient safety metrics, methicillin-resistant *Staphylococcus aureus* (MRSA) nares tracking, and regimen information. The dashboard is interactive and customizable with the ability to filter by unit, antimicrobial, and time frame and to drill down to patient level data.

**Conclusion:**

Our dashboard provides a novel tool for ASPs to continuously assess adherence to institutional treatment guidance. The data can also be used by ASPs to identify stewardship opportunities, measure the impact of CAP-related initiatives, set quality improvement targets, and quickly display information to key stakeholders.

**Disclosures:**

Lucy S. Witt, MD, MPH, Merck & Co: Grant/Research Support

